# Rapid progression of hepatocellular carcinoma in a pregnant woman: A case report

**DOI:** 10.1002/ccr3.6558

**Published:** 2022-11-06

**Authors:** Takayoshi Iijima, Soichiro Obata, Makoto Chuma, Etsuko Miyagi, Shigeru Aoki

**Affiliations:** ^1^ Perinatal Center for Maternity and Neonates Yokohama City University Medical Center Yokohama Japan; ^2^ Department of Gastroenterology Yokohama City University Medical Center Yokohama Japan; ^3^ Department of Obstetrics and Gynecology Yokohama City University Hospital Yokohama Japan

**Keywords:** cesarean section, chemotherapy, hepatocellular carcinoma, pregnancy, recurrence

## Abstract

Hepatocellular carcinoma (HCC) in pregnant women is rare; however, a recurrence of the disease is followed by rapid lesion progression during pregnancy. We experienced a case in which HCC recurred during pregnancy and rapidly worsened. After delivery at 33 weeks, she underwent chemotherapy and made a good progress.

## Graphical abstract

Hepatocellular carcinoma (HCC) in pregnant women is rare; however, a recurrence of the disease is followed by rapid lesion progression during pregnancy. Hepatologists and obstetricians should work collaboratively for early detection of recurrence.

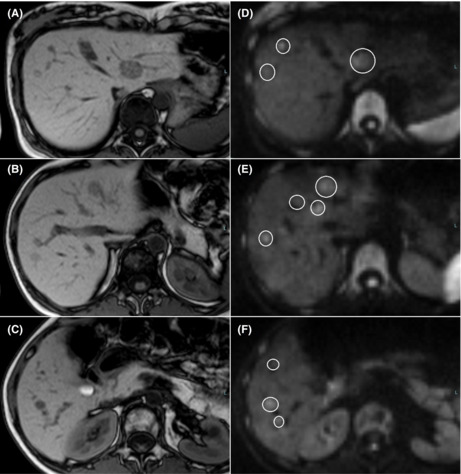

## 1 INTRODUCTION

In 2018, there were an estimated 661,000 cases of hepatocellular carcinoma (HCC) worldwide, with a female incidence rate of 3.4/100,000, which is significantly lower than the male incidence rate of 11.6/100,000.^1^ Cases of HCC detected during pregnancy have been reported, in which liver resection was performed following artificial abortion^2^ or when radiofrequency ablation (RFA) had been performed during pregnancy.^3^ There have been very few reports of recurrent HCC during pregnancy; hence, the prognosis is not well known.^3^, ^4^, ^5^, ^6^ It has been suggested that HCC may progress more rapidly and have a poorer prognosis in pregnant women than in non‐pregnant women.^7^ Whether treatment should be initiated during pregnancy or after pregnancy termination should be considered on an individual basis, depending on the gestational age and HCC progression. In this case report, we describe a patient who had HCC relapse, which showed a rapid progression during pregnancy, who was treated with chemotherapy after delivery, and who had a favorable outcome.

## 2 CASE HISTORY

**FIGURE 1:**
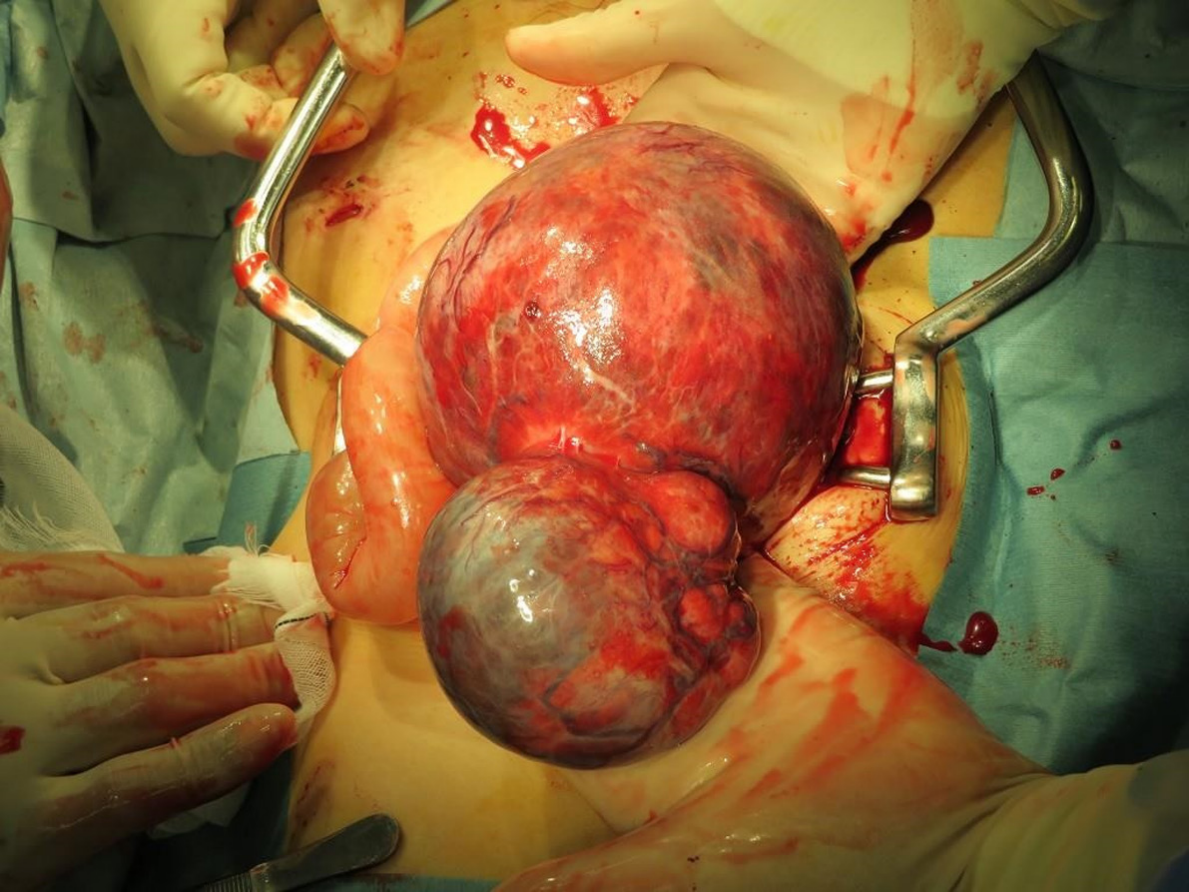
Hepatocellular carcinoma on hepatic segment 6 during surgical resection

**FIGURE 2:**
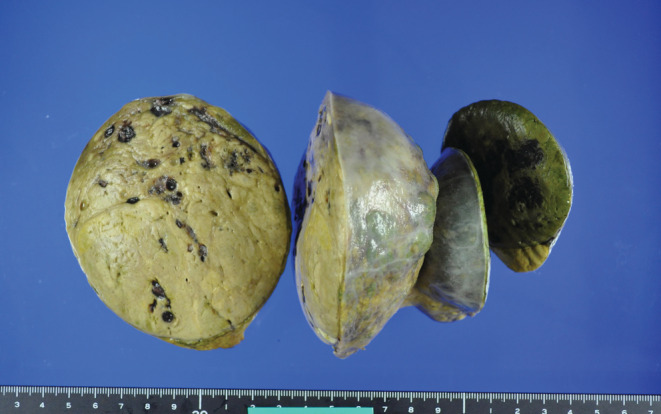
Hepatocellular carcinoma comprising 11 and 7 cm diameter lesions.

**FIGURE 3:**
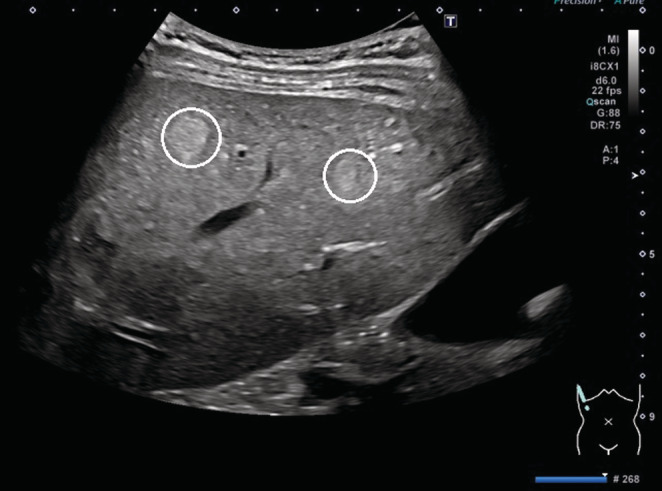
Grayscale liver ultrasonography showing nodular lesions (white circles) at 14 weeks of gestation.

**FIGURE 4:**
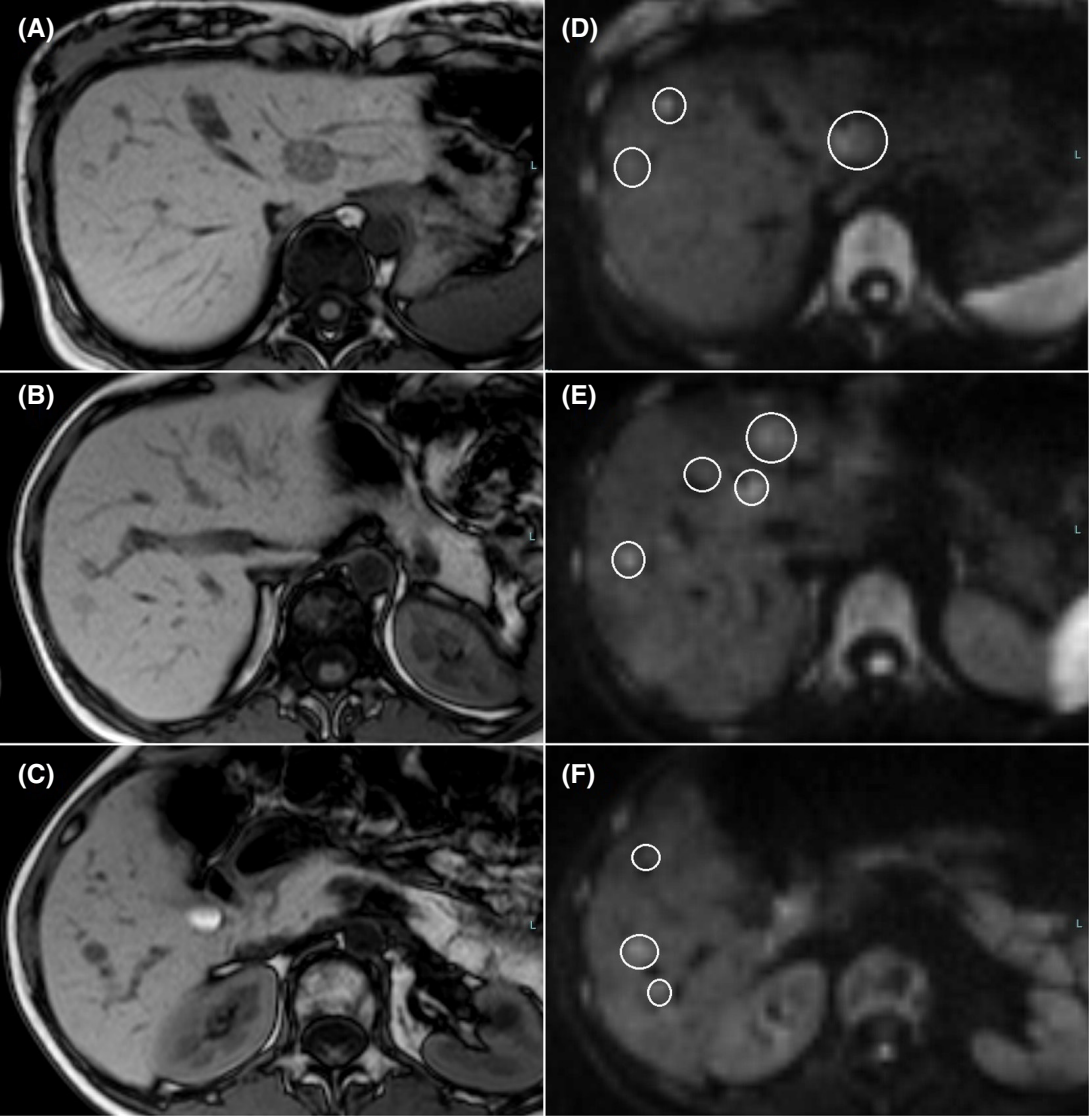
Axial T1‐weighted MRI images (A–C) and diffusion‐weighted images (D–F) show at least 10 lesions (white circles) at 30 weeks of gestation. HCC, hepatocellular carcinoma; MRI, magnetic resonance imaging.

A 40‐year‐old primipara at 30 weeks of gestation was referred to our hospital with suspected HCC recurrence. She had a history of alcohol consumption but had never previously been diagnosed with liver disease or diabetes mellitus. At 35 years old, she was diagnosed with breast cancer (noninvasive ductal carcinoma) and underwent a partial mastectomy. During the breast cancer evaluation, an intra‐abdominal mass measuring 18 cm in size was incidentally found. Initially, uterine myoma was suspected; however, laparotomy findings indicated that the tumors were liver‐derived lesions contiguous with hepatic segment 6 (S6) and were 11 and 7 cm in size. The tumors were prone to bleeding and were resected to control the bleeding. The diagnosis was HCC with extrahepatic growth. Postoperative computed tomography scans showed no residual or metastatic lesions, and the patient was TNM stage III **(**Figures 1 and 2 **)**. The liver tissue on the margins of the pathology specimen adjacent to the lesion was normal. There was no history of fatty liver disease, cirrhosis, or hepatitis, and her blood tests were negative for hepatitis B virus, hepatitis C virus, and human immunodeficiency virus infection. Three months after surgery, RFA was performed on a 14 mm‐sized recurrent lesion at S3. One year after surgery, the recurrent lesion in S4 was again treated with RFA. Stereotactic body radiation therapy was performed for lesions attached to the hepatic vein. Thereafter, the patient was referred from our hospital to another hospital that had a Gastroenterology Department but no Obstetrics and Gynecology Department. She was followed up with contrast‐enhanced magnetic resonance imaging (MRI) every 3 months at the hospital, and there were no recurrences. MRI performed 2 years and 3 months after the last treatment showed no recurrent lesions and, in the same month, she became pregnant through in vitro fertilization. A transabdominal ultrasound at 14 weeks of gestation revealed three nodules 7–10 mm in size; however, a contrast‐enhanced MRI was not conducted at the hospital because of her pregnancy **(**Figure 3 **).** At 30 weeks and 2 days of gestation, the patient was referred to our hospital, which has Gastroenterology, Obstetrics, and Gynecology departments. MRI showed >10 recurrent lesions **(**Figure 4 **)**. Due to the poor prognosis of recurrent HCC and multiple lesions that could not be treated locally, systemic chemotherapy was started immediately. After betamethasone (12 mg intramuscularly twice daily) and MgSO4 (4 g intravenously over 20 min) administration, a Cesarean section was performed at 31 weeks and 2 days of gestation. The surgery course was uneventful; there were no ascites, obvious nodules, or suspicious lesions for seeding. A male neonate weighing 1460 g was delivered and admitted to the neonatal intensive care unit. The baby was discharged at 52 days of age without major complications. The patient was discharged on the seventh postpartum day without postoperative complications. Systemic chemotherapy with lenvatinib (8 mg/day) was initiated 2 weeks after delivery. Atezolizumab 1200 mg and bevacizumab 15 mg/kg were started 1 month after the Cesarean delivery. Breast‐feeding was not recommended because of the systemic chemotherapy, and the patient agreed. Ten months post‐delivery, the patient continued chemotherapy, and no recurrence or progression of the disease was observed.

## 3 DISCUSSION

We report a rare case of HCC recurrence during pregnancy. A rapid progression of the recurrent disease was observed, and systemic chemotherapy was successfully used to control the disease progression after delivery. It has been suggested that HCC may deteriorate more rapidly during pregnancy than in non‐pregnancy and that pregnancy termination may contribute to HCC control.

We encountered an unusual case of HCC that recurred during pregnancy. To our knowledge, only four recurrent cases of HCC during pregnancy have been reported. In a review of 48 cases reported in 2011, only one recurrent case was identified.^4^ Maeda et al.^2^ reported a case of recurrent HCC at 20 weeks of gestation. An artificial abortion was performed, followed by hepatic resection, and the patient had no recurrence at 2 years. Hung et al.^5^ reported a case of recurrent HCC detected at 39 weeks of gestation. The patient underwent hepatectomy at 26 weeks of gestation and survived for more than 4 years after delivery. McCarthy et al.^6^ reported a case of HCC recurrence at 17 weeks of gestation. The patient strongly desired to continue with the pregnancy and delivered at 33 weeks of gestation despite disease progression during pregnancy. After delivery, disease progression was not observed for 6 months.

In a 2011 review, the median survival for HCC during pregnancy was 18 months for cases prior to 1995 and 25.5 months for cases reported after 1995.^4^ Treatment options include surgical resection, transcatheter arterial chemoembolization, radiation therapy, hepatic arterial infusion, and systemic chemotherapy, as in non‐pregnant patients. In this case, recurrence was suspected during a medical follow‐up at 14 weeks of gestation; however, MRI was not conducted because of the pregnancy, and the patient remained untreated until 30 weeks of gestation. At the time of referral to our hospital, there were >10 recurrent lesions identified, and local treatment was not possible. However, after delivery, chemotherapy with lenvatinib, atezolizumab, and bevacizumab was promptly initiated, and the clinical course was favorable without progression or recurrence. When a patient with a malignant tumor becomes pregnant, the Obstetrics Department along with other relevant departments must work closely together to monitor for any worsening of the patient's condition.

In this case, recurrence was suspected during an ultrasound examination at 14 weeks of gestation. At 16 weeks, the number of lesions had increased and had progressed rapidly within this short period. The possibility that pregnancy may influence HCC recurrence and progression has been frequently noted in previous reports.^7^ Immune tolerance and high estrogen status during pregnancy may promote hepatocyte division and angiogenesis; however, biological confirmation is difficult to obtain.^2^ Following a rapid spread of the disease during pregnancy, chemotherapy commenced after delivery, bringing the disease under control. This disease course suggests that pregnancy itself may have influenced HCC progression.

There is insufficient evidence regarding the safety of molecular‐targeted drugs and immune checkpoint inhibitors used against HCC during pregnancy. The median survival for inoperable, symptomatic HCC has previously been reported to be 8 weeks.^8^ However, with the recent introduction of new chemotherapeutic agents such as angiogenesis inhibitors and immune checkpoint inhibitors for the treatment of unresectable advanced HCC, more treatment options are available with improved prognosis.^9^ Furthermore, atezolizumab plus bevacizumab significantly extends overall survival compared with sorafenib, with an estimated survival rate of 67.2% at 12 months.^10^, ^11^ However, the safety of these drugs during pregnancy remains unclear. Angiogenesis inhibitors should not be used during pregnancy as angiogenesis is important for fetal development.^12^ Immune checkpoint inhibitors also increased the risk of spontaneous abortion in animal studies; however, there is limited evidence concerning their use in humans, and maternal immune‐related adverse events and risks of an increase in birth defects are unknown.^13^ If the HCC recurrence had been detected in the 20‐week gestation period, it would have been an option to continue the pregnancy and start treatment while evaluating the progression of the lesion. However, in this case, the patient was already in the 3rd trimester, and the gestational age was >30 weeks. We decided to terminate the pregnancy after betamethasone and MgSO4 administration and hasten maternal treatment after delivery. Chemotherapy may be considered while the pregnancy continues after evaluation of the gestational age; however, termination should also be considered. It is important for hepatologists and obstetricians to work collaboratively to avoid overlooking recurrent findings and decide their treatment plan.

## 4 CONCLUSION

We encountered a case of HCC that recurred during pregnancy that had remained untreated for 16 weeks. Since recurrence and metastasis in HCC cases may occur more rapidly during pregnancy than in non‐pregnancy, hepatologists and obstetricians should work collaboratively to avoid overlooking recurrent findings and decide their treatment plan. Careful follow‐up is necessary if a patient intends to carry the pregnancy to term.

## AUTHOR CONTRIBUTIONS

TI contributed to the finalization of the manuscript and performed the surgery. SO contributed to the first draft and finalization of the manuscript, determined the course of treatment, and performed the surgery. MC determined the course of treatment and performed the chemotherapy. EM and SA supervised the case report.

## CONFLICT OF INTEREST

The authors declare to have no conflicts of interest regarding the publication of this article.

## ETHICAL APPROVAL

This study was conducted ethically in accordance with the World Medical Association Declaration of Helsinki.

## CONSENT

Written informed consent was obtained from the patient to publish this report in accordance with the journal's patient consent policy.

## Data Availability

Data sharing not applicable ‐ no new data generated.
